# Global transmission pathways and adaptive molecular evolution of avian infectious bronchitis GI-23 (Variant 2)

**DOI:** 10.1038/s41598-026-63344-y

**Published:** 2026-07-27

**Authors:** Mohamed H. Houta, Giovanni Franzo, Azza A. El-Sawah, Matteo Legnardi, Kareem E. Hassan, Ahmed Ali, Claudia M. Tucciarone, Magdy F. Elkady, Mattia Cecchinato, Salama A. S. Shany

**Affiliations:** 1 Poultry Diseases Department, Faculty of Veterinary Medicine, Beni-Suef University, Beni- Suef, 62511 Egypt; 2 Department of Animal Medicine, Production and Health (MAPS), University of Padua, 35020 Legnaro, Italy

**Keywords:** Infectious bronchitis virus, Poultry, Gammacoronaviruses, Phylogeography, Selection pressure, GI-23, Ecology, Ecology, Evolution, Genetics, Microbiology

## Abstract

The infectious bronchitis virus (IBV) causes significant losses in commercial poultry egg and meat production. Since its first emergence, the genotype GI-23 (Variant 2) has progressively increased its geographical distribution, shown a significant virulence, and challenged the vaccine efficacy. Global viral transmission could be attributed to multiple factors including human related activities such as poultry and vaccine trades while other contributing factors such as the role of wild birds cannot be neglected. Therefore, A total 260 full S1 GI-23 sequences were obtained from Genebank, structured and intensively analyzed. The recombination analyses were conducted using RDP5 and GARD on the total dataset to identify the possible recombinant events. The recombinant-free dataset (214 sequences) was phylogeographically analyzed using BEAST 1.10, then selection pressure analyses to identify the possible migration routes and selection pressure markers, respectively. Recombination analyses showed multiple internal recombination events in 17.6% of the sequences. Phylogeographic analysis suggested Central and Eastern European (CEE) countries (i.e., particularly Poland and Romania) as likely sources of American GI-23-like virus outbreaks. Interestingly, the selection pressure analyses indicated positive selection signals, identifying five codons in some previously distinguished critical positions such as 61, 94, 119, 127, and 197. The virus extended geographical spread as well as molecular evolution through recombination and positive selection in the spike gene poses a major concern for increased adaptation and enhanced pathogenicity in susceptible domesticated and probably wild birds. Further studies on the impact of both recombination events and the identified positive selection codons are highly recommended to elucidate the molecular evolution mechanisms of IBV GI-23.

**Supplementary Information:**

The online version contains supplementary material available at https://doi.org/10.1038/s41598-026-63344-y.

## Introduction

Infectious bronchitis (IB) is a contagious viral disease that causes severe losses in poultry meat and egg production worldwide^1^. Since the first description of the infectious bronchitis in 1931, the virus has evolved, mutated, and recombined, along with other coronaviruses, due to the lack of proofreading activity of its RNA-dependent RNA polymerase (RdRp) and the discontinuous transcription that relies on the template-switching property of the viral RdRp^2–4^. So far, IBV has been limited to avian species; however, the primary host is chickens^5^.

The family *Coronaviridae* is classified into three subfamilies, one of which is *Orthocoronavirinae*, composed of four genera. Among these, *Gammacoronavirus* primarily infects birds, along with *Deltacoronavirus*. Within *Gammacoronavirus*, classification is further refined based on host species into different subgenera, including *Igacovirus*, *Cegacovirus*, and *Brangacovirus*. The *Igacovirus* subgenus currently includes three identified species: *Gammacoronavirus galli* (IBV), and *Gammacoronavirus anatis*^6–8^. Due to the remarkable genetic variability, IBV is currently classified according to the full analysis of the complete S1 gene into nine genotypes; the vast majority of strains fall within the genotype (GI), which is further divided into 31 genetic lineages^9^. Differences in virulence, tropism, and cross-protection have been largely demonstrated among these lineages^10–12^.

Although viral features contribute to infection outcome, other determinants, including environmental, managerial, and host-related factors, as well as co-infections, also play a pivotal role in determining the severity of the disease and its productive impact. Similarly, while IBV is an intrinsically rapidly evolving virus, its variability, as well as the features and directionality of its evolution, are influenced by host-related aspects including, but not limited to, immune selection pressure, host population heterogeneity, epigenetic factors, and host receptor selection pressure^2,13^. Additionally, environmental-related factors, including geographic and ecological factors, also impact the virus heterogeneity^14,15^. The epidemiology of IBV is strongly influenced by its interaction with vaccines from multiple perspectives. Vaccination controls virus circulation and clinical signs, but it also exerts immune pressure on the virus, which drives its evolution and selection pressure dynamics, as well as contributing to its epidemiological patterns. Other vaccination related factors contribute to its epidemiological and molecular evolution including infection occurring during vaccination, vaccine mismatch, weak immune response, low maternal-derived antibody titers, and incomplete attenuation of some live vaccine viruses with the potential for reversion to virulence^16,17^.

GI-23 (Variant 2) is among the most prevalent genotypes of IBV worldwide, ranked alongside other prominent genotypes, including GI-1, GI-13, GI-16, and GI-19^9,18^. The first isolation of GI-23 was in 1996 in Palestine. Since then, until 2010, the virus has slowly spread to surrounding countries and was first detected in Egypt in 1998; later, the virus spread to Iraq and Iran in 2008^19^. The first attempt to classify IBV isolates was mainly based on spike protein hypervariable regions (HVR) HVR1-2 and/or HVR3 and is currently recommended only for rapid diagnostics. Recently, a complete analysis of the S1 region has been required for a comprehensive characterization of isolates as recommended by Valastro et al.^20^. GI-23 generally causes high morbidity, with low or no mortality in birds older than 3 weeks, while in younger age, it causes high morbidity and high mortality (up to 30%)^21^. Postmortem examination reveals various pathological conditions including but not limited to tracheal and lung congestion, moderate intestinal swelling and kidney edema, and urates deposition in ureters^21^. Complications of infection with other pathogenic and non-pathogenic agents/factors exacerbate the infection, clinical signs, mortality rate, and persistence of the lesions^22,23^.

Since 2010, GI-23 viruses have been disseminated throughout almost all continents where chickens are densely reared such as Asia, Africa, Europe, North and South America^24,25^. Evidence-based correlation of the percentage of identity of the S1 gene and cross-protection between strains is widely accepted^26^. Despite efforts to control IBV virus through homologous vaccination or protecto-type IBV strains, the virus continued to spread to other countries and other avian hosts, causing significant losses for the poultry industry^27^. Laboratory studies of the efficacy of Protecto-type vaccines revealed variable results, with some vaccines not meeting the acceptance criteria established by the European Pharmacopeia^28^. Moreover, the current GI-23 IBV classification scheme indicates that this virus can be classified into multiple sub-lineages, and this heterogeneity may influence the cross-protection levels of homologous vaccines^29,30^.

In this study, we conducted an extensive investigation of the evolutionary dynamics of IBV on a global scale, focusing particularly on the GI-23 genotype. A detailed selection pressure analysis was performed to examine the evolutionary forces shaping IBV, with particular attention to the spike gene due to its critical role in viral infectivity, antigenicity, and immune escape mechanisms. Furthermore, we expanded the phylogeographic analysis to reconstruct the global dispersal patterns of IBV GI-23, shedding light on its possible introduction routes into the Americas. By integrating molecular epidemiology with evolutionary modeling, our aim was to provide a deep understanding of how this genotype has spread across different regions, identifying key transmission pathways and potential drivers of viral diversification.

## Materials and methods

### Datasets and phylogenetic analyses

All available Full S1 IBV GI-23 sequences (No. 260) were downloaded in August 2024 from GeneBank and identified for accession number, isolate name, country, and collection year to facilitate temporal and spatial analyses. Sequences were aligned using MAFFT v7.511 applying the Global Pairwise Alignment with Iterative Refinement (G-INS-i) strategy with two iterative cycles^31^. The full list is provided in Supplementary File 1.

### Recombination detection

 The dataset was phylogenetically analyzed using IQ-TREE to remove any sequences that might have been incorrectly classified as Variant 2. The substitution model was selected using the same software and branch support analysis was defined using both ultrafast bootstrap analysis and SH-aLRT single branch test with 1000 replicates^31^. Using recombination detection software (RDP5), the dataset was analyzed to remove potential recombinant sequences with stringent criteria to reduce false positive results, including some settings optimizations such as RDP: window size = 60, Maxchi and Chimera: percentage of variable size = 0.1, Siscan = windows 300; DSS window size 300 step size and smoothing = 10 and distance plot window size = 300. Additionally, recombinant positive signals were only included if the p-value was < 0.01 and the signal was detected by five or more methods^32^. After removal of the identified recombinant strains, alignment was cross-validated with GARD analysis using normal mode, gamma site-to-site variation with rate classes 4, to confirm the absence of residual recombination breakpoints^33^.

### Phylodynamic and phylogeographic analysis

Prior to phylodynamic analysis, the temporal signal of the cleaned, recombinant free dataset was assessed using root-to-tip regression analysis in TempEst v1.5.3. Bayesian phylodynamic analyses were conducted using BEAST v10.5.0-beta5, applying the substitution model parameters estimated by IQ-TREE to ensure consistency. A relaxed molecular clock was selected as previously proved to be effective in modeling coronaviruses rate variation among branches^25,34^. Variations in the effective viral population size were estimated using the non-parametric Coalescent SkyGrid model, setting 50 parameters and the time at least transition point to 100^35,36^.

For phylogeographic analysis, a discrete trait model (Location) coupled with Bayesian Stochastic Search Variable Selection (BSSVS) using a symmetric migration model was implemented to identify through Bayesian factor (BF) estimation, using SPREAD3, the significant (BF > 3) migration pathways between different countries^36,37^. The migration model has been efficiently applied in multiple previous studies to work with under sampled locations and dates^25,34^.

Three independent runs of 50 million generations, sampling parameters, and trees every 10,000 generations, were performed and combined using LogCombiner. The first 10% of samples from each run were discarded as burn-in. Convergence of the MCMC chains and adequate mixing were assessed using Tracer v1.7.2^38^. Subsequent analysis was performed using R studio benefitting from different libraries including (dplyr) for data grouping, summarizing, and modifying.

### Selection pressure analysis

Selection pressure analyses were conducted on the recombinant free dataset using the Datamonkey web server using four distinct codon-based methods implemented in the HyPhy package^33^. Main analyses were deployed, including MEME to detect sites undergoing episodic positive selection (i.e., selection acting on a subset of lineages), and the FEL, SLAC and FUBAR approach to estimate pervasive selection across sites. In MEME, SLAC, and FEL, a site was considered positively selected if its p-value was below 0.1 after multiple testing corrections, while FUBAR used a posterior probability threshold (commonly ≥ 0.9) to classify sites as positively or negatively selected. To ensure consistency, selection pressure sites were selected based on the agreement of the results of the four considered methods^33^. The identified features of interest were mapped onto the quaternary structure of the spike, using the 6CV0 assembly, which was downloaded from the PDB and subsequently visualized and edited in Chimera.

## Results

### Recombination analyses

The sequences collected were published between 1998 and 2024, representing 13 countries. Out of 260 sequences that were initially identified as GI-23 like strains and further verified by phylogenetic analysis using IQ-TREE, 46 (17.6%) sequences were identified as recombinant sequences using RDP5. The following GARD analysis on the dataset after recombinants removal detected no recombination breakpoint at the setting significance level, confirming the initial analysis results using RDP5. Then, the recombinant-free dataset (214 sequences), representing 12 countries, was used later for the subsequent analyses. The recombinant-free list is provided in Supplementary File 1.

### Phylogeographic analysis

A positive correlation between the sampling date and root-to-tip genetic distance was observed in TempEst analysis for temporal signal detection. The regression yielded a positive slope of 6.66 × 10⁻⁴ substitutions/site/year (*R²* = 0.005, *r* = 0.071*)*, confirming a positive trend but with a week temporal structure for molecular clock inference. The short sampling interval (2006–2024) may be insufficient to capture the full evolutionary dynamics of IBV GI-23, and the inferred divergence times should be interpreted with wide credible intervals. However, the phylogeographic migration routes, which rely on discrete trait reconstruction rather than strict molecular clock calibration, are less affected by this limitation. For phylogeographical analysis, the best-fit model, GTR + F+I+G4, was selected based on Bayesian Information Criterion (BIC) from Maximum likelihood phylogenetic analysis using IQ-TREE. After three successive BEAST runs using the selected parameters, the combined BEAST output files showed an Effective Sample Size (ESS) greater than 200 for all included parameters included in Tracer. Then, both the Maximum Credibility Tree (MCC) and the global migration map were generated using relevant packages in R studio. Interestingly, the migration map showed that the first introduction of GI-23 to South America was through Europe. For instance, migration from Poland to Brazil had a BF = 6.59, while migration from Romania to Brazil had a BF = 3.56.

**Fig. 1:**
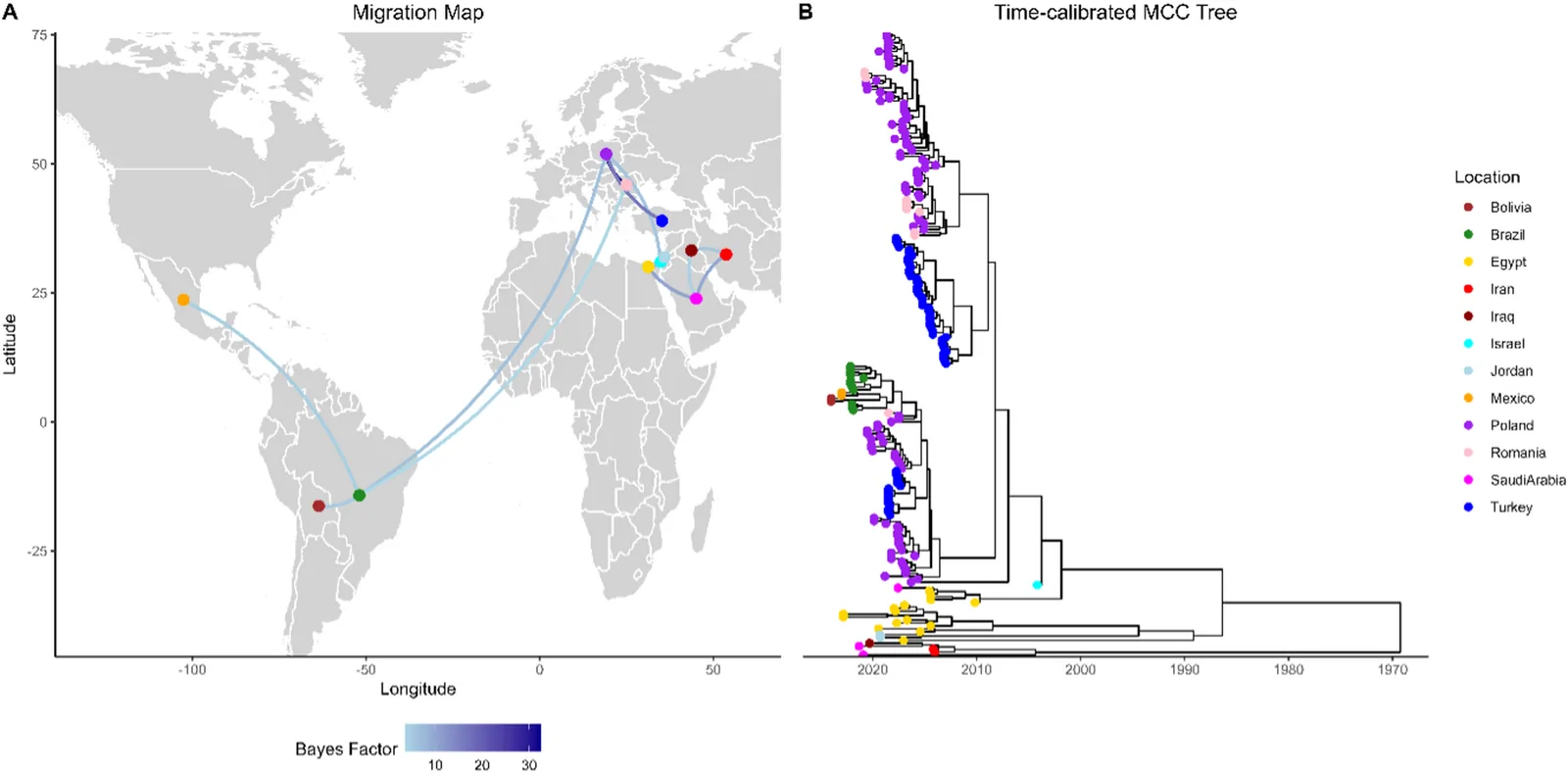
Phylogeographical analyses of IBV GI-23 identifying possible intra- and inter-continental transmission routes. (**A**) Migration map for IBV GI-23, Countries are colored based on color legend, and migration lines are bidirectional and colored according to BF. (**B**) Time Calibrated Maximum Credibility Tree for annotated based on nodes median heights and countries are colored based on the same color legend for (A) while tip labels are hidden for better visualization.

The most probable ancestral reconstruction indicates that Brazil is the first main gateway for introduction of the GI-23 and subsequently spread in South America, especially in Bolivia with BF = 5.25. However, despite the longer distance between Brazil and Mexico (North America), the migration map showed a well-supported migration signal of IBV GI-23 from Brazil to Mexico (i.e. BF = 4.78). In the old world, Egypt, Iran, Turkey, and Palestine are probably acting as an active triad for repeated IBV outbreaks and transmission of GI-23 to other neighboring countries. For instance, Egypt could play a major role in transmission with other surrounding countries including the Palestinian region, Jordan, and Saudi Arabia with BF support of 8.24, 6.53, and 10.65, respectively. In addition, Iran showed a similar pattern to that of the surrounding countries, including Iraq and Saudi Arabia with BF support of 5.86 and 10.05, respectively. Turkey may act as as an entry gateway to Europe with high BF support = 16.48 on the migration route to Poland as shown in Fig. 1A.

The time-calibrated MCC tree showed that the clustering of IBV GI-23 is correlated to the countries and/or regions. For instance, the most prevalent subclade in Europe, North and South America is IS/1494/06-like strains (GI-23.1), while in the Middle East, the most prevalent subclade is EG/Beni-Suef/01-like strains (GI-23.2). Only Israel showed a single detection of IS/Variant2/98 (GI-23.3) with no repeated detection with complete S1 gene sequencing and characterization, as illustrated in Fig. 1B.

### Selection pressure analyses

The analysis of selective pressure on the spike (S) gene of the IBV GI-23 lineage employed an extensive array of evolutionary methodologies, including SLAC, FEL, MEME, and FUBAR. Each of these approaches evaluates the selective pressure through a quantitative assessment of synonymous and nonsynonymous substitutions, offering distinct parameters that elucidate the nature and significance of selection exerted on codon sites. The application of these four methods revealed five potential codons that exhibit strong evidence of positive selection. The statistical support is shown in Table 1.

**Table 1:** Selection pressure summary for the infectious bronchitis virus GI-23 using hyphy package.

Codon	SLAC	FEL	MEME	FUBAR	Substitutions	Count
61	0.0277	0.0784	0.039	0.946	S→P	4
					P → T	1
					P → S	1
					S → T	1
					S → L	1
94	0.000917	0.0196	0.039	1.000	V→ A	11
					T → A	5
					T → U	5
					A → T	3
					V → T	2
119	0.00977	0.0392	0.02	0.977	P → P	13
					H → P	6
					R → R	3
					F → F	3
					S → S	3
127	0.0439	0.0784	0.078	0.950	F → F	5
					L → M	3
					L → Q	2
					L → P	1
					L → L	1
197	0.0559	0.0196	0.059	0.998	S → K	1

**Fig. 2:**
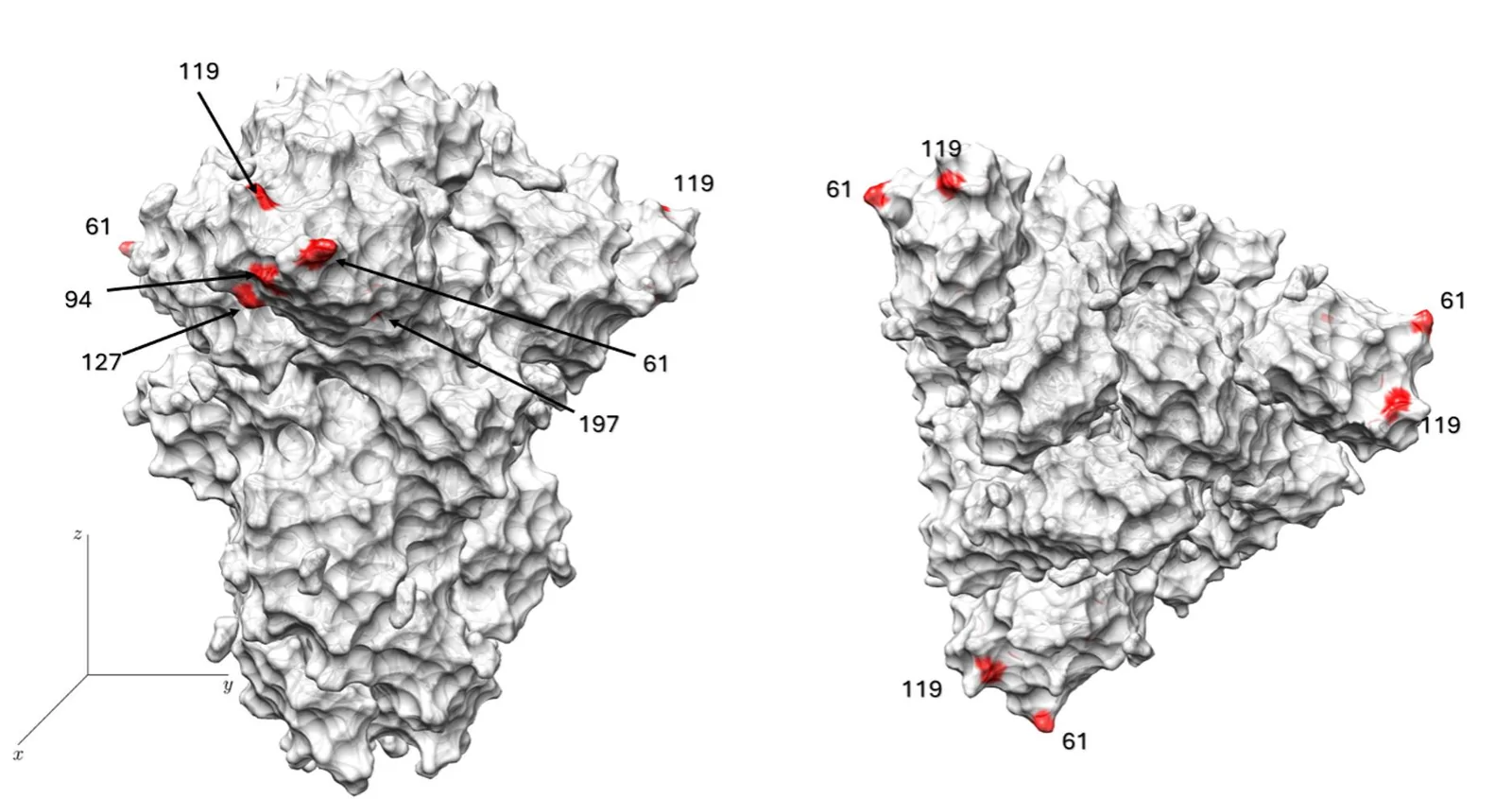
Quaternary structure of the IBV Spike trimer reconstructed on ID: 6CV0 3D structure of infectious bronchitis virus Spike protein. The front (left) and upper (right) views of the protein are shown. The sites identified as undergoing positive selective pressures in the present study are highlighted in red, with the corresponding amino acid positions labeled on all visible subunits.

The SLAC method evaluates selection by comparing nonsynonymous (dN) to synonymous (dS) substitution rates, with significance set at *p* ≤ 0.1. Positive selection was detected at codons 61 (*p* = 0.0277), 94 (*p* = 0.000917), 119 (*p* = 0.00977), and 127 (*p* = 0.0439). Codon 197 (*p* = 0.0559) was near significance, highlighting selective pressures on these sites. With the FEL positive selection was detected at cordons 61 (*p* = 0.0784), 94 (*p* = 0.0196), 119 (*p* = 0.0392), 127 (*p* = 0.0784), and 197 (*p* = 0.0196), indicating adaptive evolution. The MEME method also detected episodic diversifying selection at codons 61 (p-value: 0.039), 94 (p-value: 0.039), 119 (p-value: 0.02), 127 (p-value: 0.078), and 197 (p-value: 0.059). The FUBAR method detected pervasive positive selection using a Bayesian approach at codons 61 (PP = 0.946), 94 (PP = 1.000), 119 (PP = 0.977), 127 (PP = 0.950), and 197 (PP = 0.998), indicating robust selective pressure. The analyses identified that codons 61, 94, 119, 127, and 197 in the spike gene of IBV GI-23 are subject to substantial positive selection of varying intensities, both pervasive and episodic in nature. The sites detected under positive selection in the S1 gene are visualized in Fig. 2.

## Discussion

IBV GI-23 (Variant 2) is currently circulating in Africa, Asia, Europe, and America, causing severe economic losses in poultry production. The prevalence of GI-23 was localized in some neighboring countries in the Middle East from 1996 until 2010, then the virus expanded its geographical locations to other regions in Asia, Europe, and Africa between 2010 and 2020, and later on, from 2020 to 2025, the virus was detected in South and North Americas^18,24^. The IBV GI-23 was not detected in commercial chicken flocks only but also in wild birds such as *Passeriformes*, *Galliformes*, *Anseriformes*, and *Pelecaniformes*^39^.

GI-23 viruses form a heterogeneous group; classical sub-lineages such as GI-23.2 (GI-23.22.1, GI-23.22.2, GI-23.2.3) include historical viruses such as EG/Beni-Suef/01, EG/1212B/12, and EG/QENA31/17, respectively, which are dominating in Egypt, GCC and Persian countries. However, the remaining countries including Israel, Turkey, European, and American countries show the highest prevalence of GI-23.1 represented by the classical strain IS/1494/06.

Traditionally, IBV was thought to be transmitted not only through human related activities such as vaccines and poultry trade but also, migratory birds were suggested to play a crucial role in inter- or intra-regional and intercontinental transmission^6,40^. In a previous study, human related activities including poultry trade between Middle Eastern countries were suggested to play a role in the intra-regional migration of IBV GI-23. Additionally, poultry trade might played an important role in the inter-regional transmission of IBV GI-23 from Egypt, Iran, and Turkey to neighboring countries in Africa, Asia, and Europe, respectively^25^. Furthermore, poultry trade is suggested to play a role in the intercontinential tranmission after detection of recombinant strain that genetically derived from Korean GI-19 and Brazilian GI-11 in Korea^41^.

Human related activities including vaccine trade may contribute to the endemicity of IBV GI-23 viruses in Europe^16^. In Africa, countries such as Libya in 2012, Nigeria in 2013, and South Africa in 2015 documented the presence of GI-23^42–44^. The viral dispersal in some African countries is controversial due to the vast distance of the identified countries, contributing to the probable role of human related activities such as vaccine trade in virus introduction to those countries. Interestingly, Phylogenetic analyses revealed a close relation between a GI-23.1 vaccine and the detected viruses in those countries^42,43^.

Conversely, The Great Rift Valley migratory flyway has previously been suggested to play a role in the transmission of IBV GI-23 between countries in the same region (i.e., Asia, Africa, and Europe)^6^. This migratory route appears to show a similar pattern with the epidemiological patterns observed across these countries during the initial detection periods in each respective country. For instance, the virus was initially identified in the Palestinian region in 1998 as the progenitor region of the GI-23 strain, followed by its detection in Egypt in the same year, then subsequently in Iraq and Iran in 2008, Jordan and Lebanon in 2009, Turkey in 2010, Kazakhstan in 2015, Russia in 2016, and Afghanistan in 2016. It is noteworthy that the virus was not identified in Syria before 2018, possibly due to socio-political issues from 2010 to 2025^6,18,45^. Other Asian countries, specifically members of the Gulf Corporation Council (GCC), including Saudi Arabia, Oman, and Kuwait, reported cases in 2009, whereas the United Arab Emirates did so in 2022, likely reflecting the influences of the migratory path^46,47^. In Europe, the first detection incidents were observed in Ukraine, Lithuania, and Armenia in 2015, followed by Romania in 2016, while other countries such as Germany reported the virus in 2021^24^.

In the current study, the emerged transatlantic transmission of GI-23.1 from Europe to South America was reconstructed using BEAST phylogeographic analysis, which supports the previous note that Brazilian GI-23 like viruses are grouped with Polish viruses^48^. Such a connection is difficult to explain due to the vast geographical distance and the clear geographical barriers separating the two regions. While human related activities such as poultry or vaccine trade could be speculated as a root cause for IBV GI-23 introduction to South America, migratory birds could not be also neglected due to the growing evidence for wild migratory birds’ contribution in IBV epidemiology^49^. The role of migratory birds in global transmission of IBV GI-23 is quite questionable due to multiple factors such as ecological and molecular epidemiological data limitations. For instance, the ecological data until now is insufficient including but not limited to both active and passive surveillances in wild and domesticated animals and/or birds. Moreover, the molecular epidemiological data is limited due to sampling bias as well as limited availability of full S1 genetic data.

Regardless of the potential transmission pathways, the phylogeographical analysis suggested Central and Eastern European countries (CEE) including Romania as a dispersal hub for global IBV transmission. On the other hand, Brazil, likely point of entry to the Americas, played a pivotal role in further viral spread in the Americas. Bolivia, Mexico, and Uruguay were subsequently affected by GI-23 viruses that are closely related to Brazilian isolates^50,51^. Interestingly, Brazil is a major poultry worldwide exporter, including genetic lines and day-old chickens. Recently, new markets have opened, particularly in American countries such as Mexico, Uruguay, and other South American countries, justifying the inferred links^52,53^.

From the global epidemiologic perspective to the molecular perspective, IBV has a complicated evolutionary process driven by diversity, recombination, and selection pressure^2^. Diversity is mainly manifested due to their nested replication, large genome size, minimal proofreading capabilities associated with the RdRp, while recombination is mediated by a copy-choice mechanism^54,55^. However, selection pressure is a fundamental evolutionary force that drives genetic and phenotypic changes in viral populations.

The recombination analyses revealed a high prevalence of recombinant isolates among IBV GI-23 isolates (17.6%), a similar pattern had been identified previously^25^. Recombination events in full S1 may impact viral fitness as well as vaccine efficacy^49,54,55^. For instance, an increase in the viral fitness may facilitate interspecies transmission and increase the IBV virulence. Meanwhile, the efficacy of the vaccine may be reduced due to the introduction of novel genetic elements. These elements could reduce the genetic similarity between the vaccine and newly generated recombinant strains and consequently alter one or more pivotal neutralization epitopes^49,54,55^.

Across the full S1 gene, five codons have been identified as positive selection in all test methods (i.e., FEL, FUBAR, MEME, and SLAC) spread across multiple regions. Codon 61 is one of the most significant codons due to its location in the receptor binding domain (RBD), hypervariable region 1 (HVR1), N-terminus domain (NTD), and the critical amino acids that have been previously identified for respiratory tissue tropism. Interestingly, this codon is closely located beside one of the most critical N-glycosylation sites (59 aa)^56^ and to one of the cross-protective epitopes that have been recently verified (67–82 aa)^57^. Overall, previous studies suggested that position 61 has an impact not only on the affinity to respiratory tissues but also cross-protective response and immune escape due to its location adjacent to an important glycosylation site^56–59^.

In the HVR2, positions 94, 119, and 127 were also detected, whereas the region is suggested in some previous studies to play a crucial role in renal tropism in QX strains (99–159 aa) as well as in the cross-protective epitope (83–96 aa) that have been identified in 4/91 to be protective against M41 and QX^57,58^. Moreover, a recent study verified an N-glycosylation position in this region at amino acid position 126; therefore, the nearby 127 aa was reported under selective pressure in the present study^56^. The intensive presence of positive selection markers (3 positions) in this HVR2 could be correlated with the increased nephro-pathogenicity of GI-23 in comparison to other genotypes as well as immune evasion due to alteration in glycosylation and lowered field efficacy of protecto-type vaccines^21,29^. This theoretical correlation needs laboratory testing for confirmation.

A comparable pattern was found for position 197, which was located near a recently identified N-glycosylation site 194^56^. Therefore, considering the role of glycosylation in spike functionality and its ability to shield against the immune response, this evidence could also suggest potential immune evasion mechanisms.

## Conclusion

The IBV GI-23 showed increased geographical distribution and possibly enhanced transmission mechanisms on the global scale, which might involve human related activities including poultry or vaccine trades. Moreover, continued efforts are required to improve active surveillance of wildlife infectious diseases and the role of wildlife in the transmission of IBV viruses not only between inter- and intra-regional but also intercontinental. Another major concern is the continuous evolution of the virus through recombination, as discussed in the article, along with the persistent occurrence of mutations and selection pressure at some identified critical sites in the spike gene. These sites are previously distinguished as functionally and biologically important codons for viral predominance and survival. Further studies are highly recommended to elucidate the role of wild birds in infectious bronchitis inter- and intra-regional and intercontinental transmission and to evaluate the potential impact of the identified positive selection codons through laboratory investigations.

## Supplementary Information

Below is the link to the electronic supplementary material.

Supplementary Material 1

## Data Availability

Data is provided within the manuscript or supplementary information files.
